# One-Shot Full-Range Quantification of Multi-Biomarkers With Different Abundance by a Tandem Giant Magnetoresistance Assay

**DOI:** 10.3389/fchem.2022.911795

**Published:** 2022-05-19

**Authors:** Fanda Meng, Lei Zhang, Jie Lian, Weisong Huo, Xizeng Shi, Yunhua Gao

**Affiliations:** ^1^ Department of Clinical Laboratory Medicine, The First Affiliated Hospital of Shandong First Medical University & Shandong Provincial Qianfoshan Hospital, Shandong Medicine and Health Key Laboratory of Laboratory Medicine, Jinan, China; ^2^ School of Clinical and Basic Medicine, Shandong First Medical University & Shandong Academy of Medical Sciences, Jinan, China; ^3^ Department of Chemistry and Chemical Engineering, Chalmers University of Technology, Gothenburg, Sweden; ^4^ Shenzhen Bosh Biotechnologies, Ltd., Shenzhen, China; ^5^ College of Criminal Investigation, People’s Public Security University of China, Beijing, China; ^6^ Key Laboratory of Photochemical Conversion and Optoelectronic Materials, Technical Institute of Physics and Chemistry, Chinese Academy of Sciences, Beijing, China; ^7^ University of Chinese Academy of Sciences, Beijing, China

**Keywords:** ‘hook’ effect, multi-biomarker detection, giant magnetoresistance, biosensor, POCT

## Abstract

In this study, we reported a tandem giant magnetoresistance (GMR) assay that realized the one-shot quantification of multi-biomarkers of infection, C-reactive protein (CRP) with procalcitonin (PCT), and neutrophil gelatinase-associated lipocalin (NGAL), all of which could cover their clinically relevant concentration ranges under a different principle. In the presence of co-determined assay, we quantified these three biomarkers in undiluted human blood serum in a single test. The tandem principle, based on which quantification of CRP occurs, combines a sandwich assay and an indirect competitive assay, which allows for the discrimination of the concentration values resulting from the multivalued dose-response curve (‘Hook’ effect), which characterizes the one-step sandwich assay at high CRP concentrations. However, the entire diagnostically dynamic range, in the quantification of PCT and NGAL, was achieved by differential coating of two identical GMR sensors operated in tandem and by combining two standard curves. The sensor quantified low detection limits and a broader dynamic range for the detection of infection biomarkers. The noticeable features of the assay are its dynamic range and small sample volume requirement (50 μL), and the need for a short measurement time of 15 min. These figures of merit render it a prospective candidate for practical use in point-of-care analysis.

## 1 Introduction

The levels of C-reactive protein (CRP), neutrophil gelatinase-associated lipocalin (NGAL), and procalcitonin (PCT), which have different abundance in blood, show good responses to inflammation (Yavuz et al., 2014; Zhu et al., 2014; Nielsen et al., 2021). In this era of rising antimicrobial resistance, infection biomarkers are gradually becoming a convenient and effective tool to guide the antimicrobial use (Nielsen et al., 2021).

In clinical settings, combinations of multi-biomarkers have outperformed single biomarkers for early diagnosis of the disease (Lian et al., 2013; Bhide et al., 2019; Muinao et al., 2019), especially the combined detection of infection markers could define the precise level of inflammation. But there is a giant challenge that lies in the multi-detection caused by their different abundance and dynamic range, which also limits the widespread use of the one-shot detection of multi-biomarkers.

CRP is always used in combination with low abundance biomarkers in clinical settings (Buch and Rishpon, 2008; Pultar et al., 2009; Kilpatrick et al., 2012; Pay and Shaw, 2019); the dilution of serum is a serious problem indeed in multi-biomarker detection in one shot, where the dynamic ranges of different analytes are very different. Dilution would prevent the detection of low abundance biomarkers, and avoiding it is one of the primary concerns. Typically, detection of CRP in the undiluted human serum is usually limited by the ‘Hook’ effect (Byun et al., 2013; Oh et al., 2014; He et al., 2022), which also limits the combination with other biomarkers. In our previous work (Meng et al., 2021a), a competition assay was utilized as a complementary assay as an indicator to monitor the ‘Hook effect’ curve, so the ‘Hook’ effect curve could be utilized as the standard curve which actually contains two standard curves.

PCT and NGAL are always combined with CRP in clinical settings to predict and diagnose infection levels (Zhu et al., 2014). For the biomarkers of PCT and NGAL with low concentrations in serum, the detection method should be with high sensitivity while covering the concentrations relevant for infection diagnosis. Two sensors in tandem in one assay system were utilized to widen the detection range of N-terminal pro-B-type natriuretic peptides in our previous work (Meng et al., 2021b), which also maintain the good sensitivity. Differential coating of two identical GMR sensors operated in tandem and combining two standard curves could be ultimate in detecting PCT and NGAL biomarkers. In the tandem assay, two pairs of antibodies of different affinities to the specific biomarker were used simultaneously in one assay, in which two signals were applied to quantify the bio target.

The quantification principle of the GMR sensor is based on the change in resistance when the local magnetic field is changed due to magnetic materials binding to the surface (Nesvet et al., 2021). In the GMR assay, magnetic nanoparticles (MNPs) are always used as labels to create a magnetic field that is detected by the GMR sensor, which could realize real-time detection. Compared to traditional optical detection, the GMR biosensor has been shown to have high sensitivity and specificity, which also retained low costs and rapid testing time in bio-analysis assays (Gani et al., 2019; Ravi et al., 2022).

With these figures of merit, we developed a multiplexed quantitative platform for the detection of CRP, PCT, and NGAL, which are all bacterial infection biomarkers in clinical settings. The one-shot GMR chip realized accurate detection of infection, which also provides valuable clinical information and could aid in infection management.

## 2 Materials and Methods

### 2.1 Regents and Materials

All reagents used in this work were of analytical grade. NaHCO_3_, Na_2_CO_3_, Na_2_HPO_4_, NaH_2_PO_4_, KCl, NaCl, and BSA were purchased from Sigma-Aldrich (Merck). Tween 20 was purchased from AMRESCO (United States). NHS-biotin was purchased from Thermo Fisher Scientific, Inc. (United States). Streptavidin-conjugated magnetic particles (MNP, 100 nm) were purchased from Ademtech (France). A sample of polystyrene-grafted maleic anhydride (PS-g-MA, graft ratio 17%) was provided as a free sample by Longjia Plastics Fabrication (Jilin, China). Heterophilic blocking reagent (HBR1) was obtained from Scantibodies Laboratory, Inc. (United States). Heterophilic immunoglobulin elimination reagent (Fapon Block: HIER-E-010) was obtained from Fapon Biotech Inc. (China).

Anti-human CRP antibodies (capture antibody: MCP01 and detection antibody:MCP02) and CRP antigen were purchased from Hangzhou Yibaixin Biotechnology Co., Ltd. (China). Anti-human PCT antibodies (capture-detection antibody: pair I: MPT33-MPT34 and pair II: MPT31-MPT34) and PCT antigen were purchased from Hangzhou Yibaixin Biotechnology Co., Ltd. (China). Anti-human NGAL antibodies (capture-detection antibody: pair I: 1F2-1G2 and pair II: 4H11-1G2) and NGAL antigen were purchased from Fantibody Co., Ltd. (China). Undiluted clinical serum samples were received as a donation from the Zhujiang Hospital of Southern Medical University (China). The detection antibody was biotinylated using NHS-biotin (Zhang et al., 2015).

**FIGURE 1:**
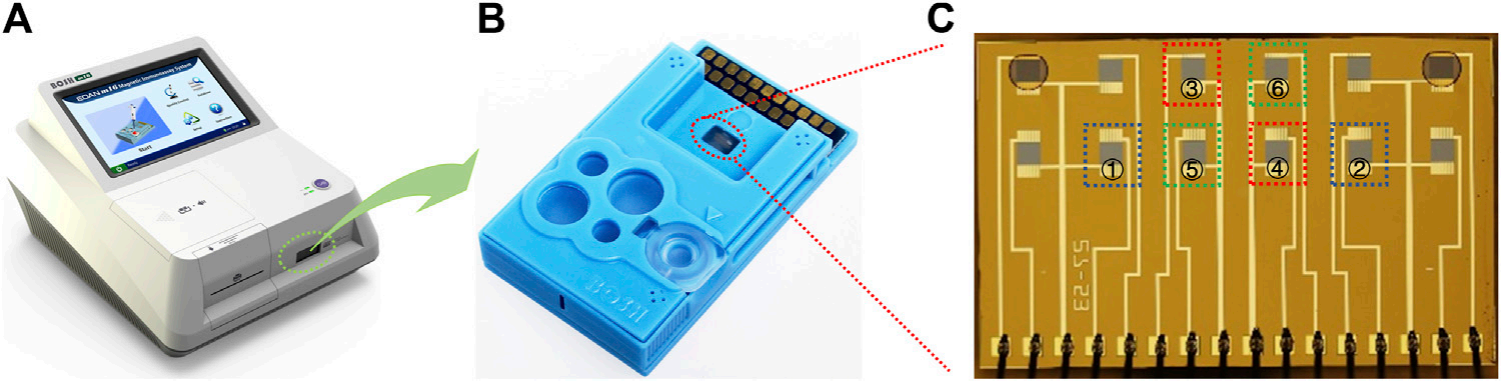
GMR immunoassay analyzer **(A)**, the polymer assay cartridge **(B)**, and the GMR sensor array chip **(C)**. The two selected sensor units used for CRP detection are marked in blue squares (① and ②). The two selected sensor units used for PCT detection are marked in red squares (③ and ④). The two selected sensor units used for NGAL detection are marked in green squares (⑤ and ⑥).

The GMR immunoassay analyzer (Bosh M16) (Figure 1A) and the polymer assay cartridge (Figure 1B) were manufactured by Shenzhen Bosh Biotechnologies, Ltd. (China).

### 2.2 Giant Magnetoresistance Chip Preparation

**FIGURE 2:**
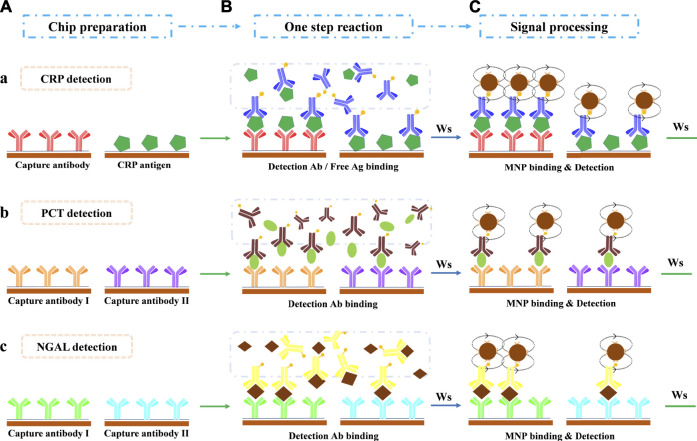
Assay protocol for multi-biomarker detection in a one-shot chip. **(A)** Chip preparation. **(B)** One-step antibody-antigen reaction after sample loading. **(C)** Magnetic particle binding and detection. Ws: washing step. **(a)** CRP detection: the combination of sandwich assay and indirect competitive assay. **(b)** PCT detection: the combination of two sandwich assays with two antibody pairs that share the same detection antibody. **(c)** NGAL detection: the combination of two sandwich assays with two antibody pairs that share the same detection antibody.

The GMR biosensor arrays comprise 2 × 6 sensors (Figure 1C). For the quantification of all three biomarkers, two sensors were selected in combination to realize each biomarker assay (Figure 1C). The detection of CRP (Figure 2A) combines a sandwich assay and an indirect competitive assay, which utilizes the multivalued dose-response curve (‘Hook’ effect) to realize the whole-range quantification of CRP. The detection of PCT (Figure 2B) and NGAL (Figure 2C) were all realized by a combination sandwich assay, which used two pairs of antibodies with different affinities to widen the detection range.

The GMR chip preparation procedure was described in our previous work (Meng et al., 2021a). The CRP, PCT, NGAL capture antibody, and CRP antigen solutions (50 μg/ml) were deposited onto the selected sensors. The antibody/antigen could be covalent to the sensor surface (Meng et al., 2016).

### 2.3 Immunoassay Procedure

For quantification of multi-biomarkers, six sensors were selected in the tandem assay (Figure 1C). Two sensors, combining a sandwich assay and competitive assay (Figure 2A), realized the CRP detection. In the one-step reaction, the sandwich assay forms a ‘Hook’ effect curve in the whole-range concentration of CRP, whereas the competitive assay could monitor the reaction in combination with the sandwich assay. The combination of these two assays makes the ‘Hook’ effect curve a possible standard curve, which extremely expanded the quantification range of the sandwich assay.

For PCT (Figure 2B) and NGAL (Figure 2C) detection, two sensors were combined for each, which realized two different affinity sandwich assays at the same time. The ultimate two pairs of antibodies with different affinities, which with a shared detection antibody, broadened the detection range and ensured the detection sensitivity as well.

As described in our previous work (Meng et al., 2021a) on the assay protocol, 50 μL sample or standard was pipetted into the sample port of the cartridge in the first step. Then, the cartridge was inserted immediately into the detector to autorun the immune reaction and to obtain the results in 15 min. The sample/standard first re-dissolved the freeze-dried detection antibodies and reacted to form the antigen-antibody complex simultaneously (Figure 2B). Washing steps by PBST (10 mM, pH = 7.4, 0.5% Tween 20) were applied to remove the extra sample/regents. Afterward, the mixture was pumped to flow through the sensor surface (flow back and forth 5 min at RT), where the free antigen/detection antibody reacted with the capture antibody/antigen and was captured. Subsequently, the carbonate buffer (0.1 M, pH = 9.6, 0.05% Tween 20, and 10% BSA) was pumped to dissolved freeze-dried avidin-coated MNPs and initiated to react with the captured biotin-modified detection antibody (5 min at RT, Figure 2C). The captured MNPs on the specified sensors were finally detected by the GMR detector, and the results were reported automatically.

### 2.4 Fitting Software and Algorithm

All standard curves were fitted by the four-parameter logistic model (y = A_2_+(A_1_-A_2_)/(1+(x/x_0_)^p)) (O'Connell et al., 1993), by Origin 9.1 software.

### 2.5 Data Processing

For the establishment of standard curves of all three biomarkers, each concentration point of the mixed standard was measured four times. A total of 91 clinical samples were analyzed, and measurement was performed once per sample.

**FIGURE 3:**
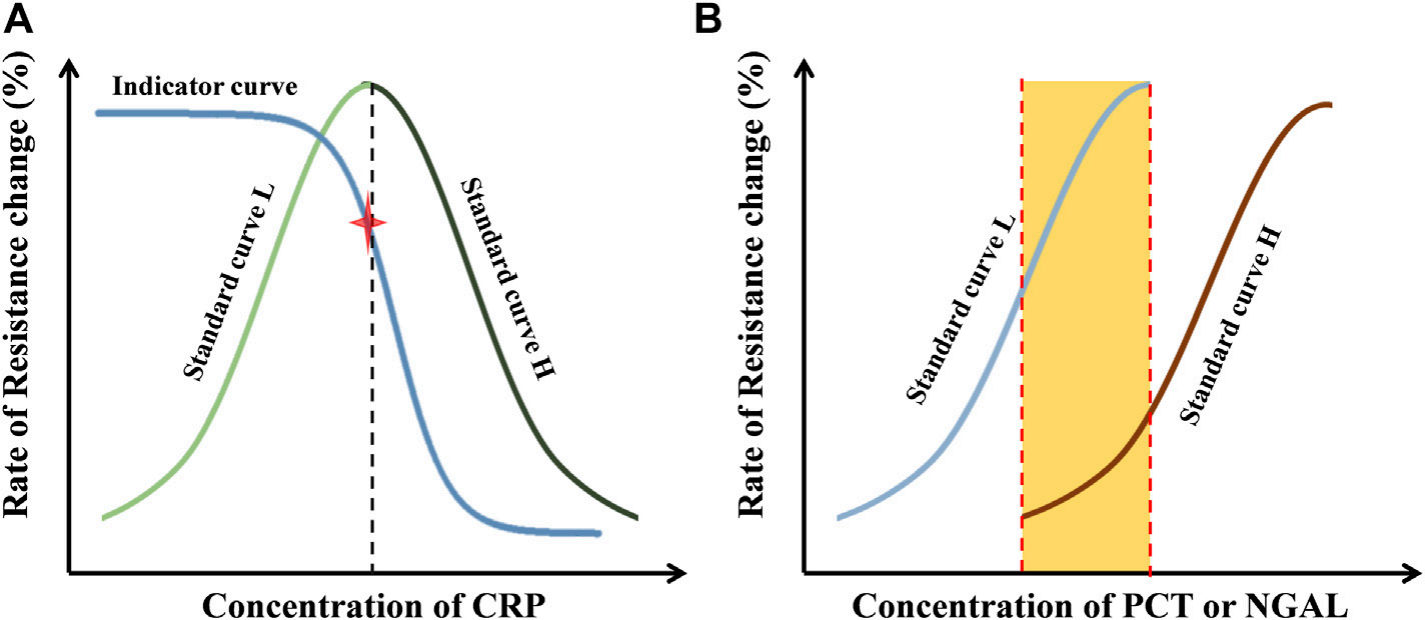
Principle of data processing. **(A)** Standard curves and indicator curves of CRP obtained with external standards from sensor ① and ②. **(B)** Standard curves of PCT (sensor ③ and ④) or NGAL (sensor ⑤ and ⑥) obtained with external standards.

In the evaluation of CRP (Figure 3A), signals on sensor ① formatted a ‘HOOK’-shaped curve, which covered the low (standard curve L) and high (standard curve H)-concentration situations. Signals on sensor ② formatted the indictor curve, which contains an indicator point to measure whether the ‘Hook’ effect occurs or not. The indicator is not used in quantification. In the real sample measurement, if the value on sensor ② is higher than the indictor point, the value on sensor ① would be calculated with standard curve L. Otherwise, the standard curve H would be appropriate in quantification.

In the evaluation of PCT or NGAL (Figure 3B), two standard curves covering the low (standard curve L) and high (standard curve H)-concentration situations were obtained, which extremely expanded the detection range. The standard curve L was used in low-concentration situations; in the other case, the standard curve H was used. The quantification in the middle range (the orange area in Figure 3B) was determined by calculating the mean of the data from both standard curves.

## 3 Results and Discussion

### 3.1 Specificity of the Assay

#### 3.1.1 Interference of Three Markers

**FIGURE 4:**
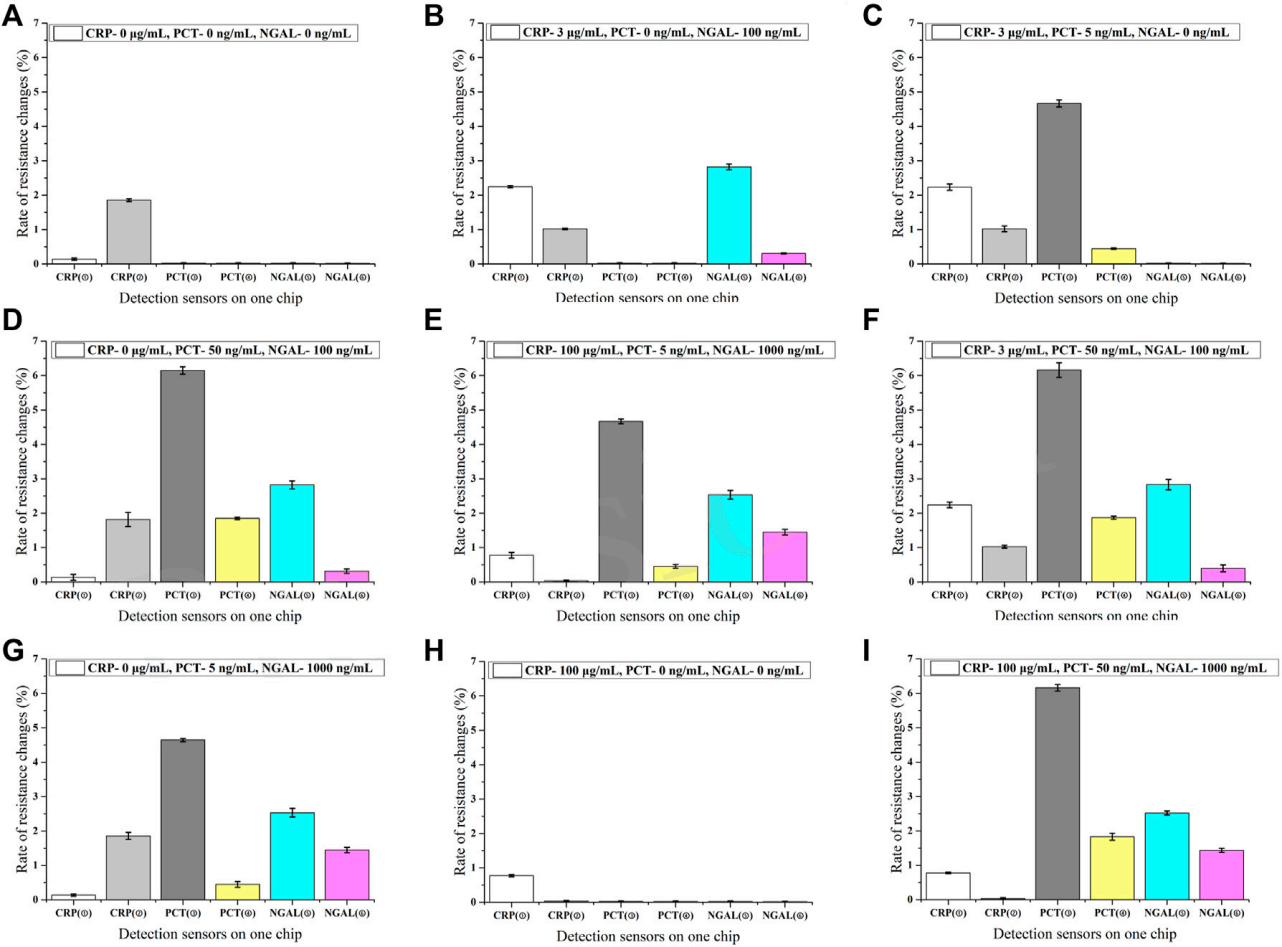
Specificity of the assay (*n* = 3) **(A–I)**. ①-⑥ in the horizontal axis represents different sensors labeled in Figure 1.

The specificity of the antibody pairs is the key factor in the multi-detection assay. The interference of the detection of three markers was evaluated at a crossing concentration (Figure 4). It shows that the combinational sensors designed for detecting the same biomarker presented undifferentiated signals at varying concentrations of two other markers. So it proves that there is no interference during the combined detection of three markers, which indicates that the assay has good specificity.

#### 3.1.2 Interference of Substances

**FIGURE 5:**
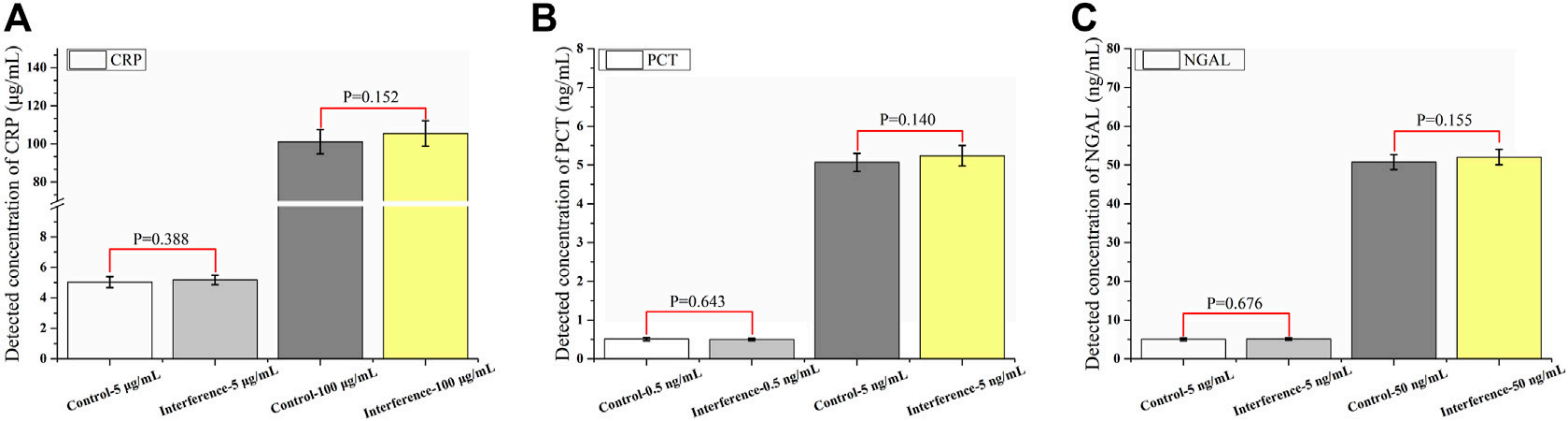
Specificity of the assay under the interference of substances (*n* = 10) **(A–C)**.

Many ingredients, especially some big proteins, in the human serum may result in nonspecific binding. To further verify the specificity of the assay, a mixture of interfering substances (1,000 μg/ml hemoglobin, 60 μg/ml bilirubin, 1,000 μg/ml triglycerides, and 1200 IU/ml rheumatoid factor) was added to the sample. Two levels (CRP: 5 and 100 μg/ml; PCT: 0.5 and 5 ng/ml; and NGAL: 5 and 50 ng/ml) were evaluated for each biomarker (Figure 5), and the results showed that there was no significant difference after the substances were added. It also proved the function of the commercial blockers we added to the assay, which helped keep the excellent specificity of the assay.

### 3.2 Precision and Long-Term Stability of the Assay

The precision was calculated by the measurements (*n* = 80), which were split into two concentration levels (Table 1). The measurement period was 20 consecutive days with two measurements per day and one concentration level. The assay cartridges used were newly fabricated on the day of detection. Measurements on the same day were denominated as intra-assay, when all other measurements were denominated as inter-assay. The RSD all under 10% shows that the precision was excellent.

**TABLE 1:** Precision of the assay (*n* = 80).

Marker	Concentration	Mean	Intra-assay Precision		Inter-assay Precision	
			SD	RSD/%	SD	RSD/%
CRP	5 μg/ml	5.012	0.437	8,71	0.465	9.28
	100 μg/ml	99.41	8.485	8,54	7.359	7.40
PCT	0.5 ng/ml	0.504	0.032	6.30	0.038	7.48
	5 ng/ml	5.036	0.313	6.22	0.305	6.06
NGAL	5 ng/ml	5.406	0.370	7.34	0.480	9.51
	50 ng/ml	49.727	1.991	4.00	1.954	3.93

**FIGURE 6:**
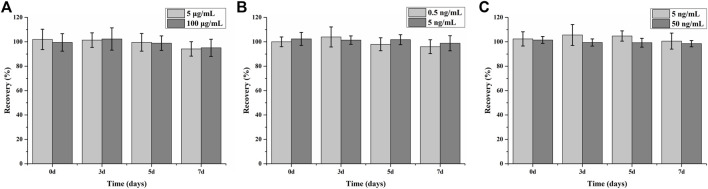
Long-term stability of assay for CRP **(A)**, PCT **(B),** and NGAL **(C)** detection (*n* = 6).

The long-term stability was investigated by incubating the fabricated cartridge at 37°C over 7 days (Figure 6). The determined recovery >94.2% and RSD <10% (*n* = 6) indicate that the assay can maintain good function for several months at 4°C. The antibody/antigen deposited onto the selected sensors was sufficient to bind to the surface; the excessive antibody/antigen on the outside could work as a protector to keep the activity of the covalently captured antibody/antigen. The method of freeze-drying we used in the assay could also greatly protect the activity of protein reagents.

### 3.3 Establishment of Standard Curves

**FIGURE 7:**
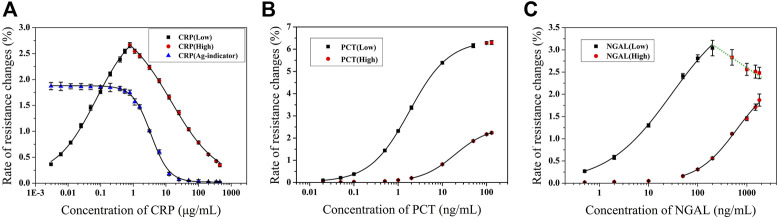
Standard curves for CRP **(A)** (Adapted with permission from (Meng et al., 2021a) Copyright ^©^ 2021 Fanda Meng, etc. Published by American Chemical Society), PCT **(B),** and NGAL **(C)** detection (*n* = 4).

The relationships between the rate of resistance change and concentration were calculated to establish the standard curves. All standard curves were fitted by the four-parameter logistic model (y = A_2_+(A_1_-A_2_)/(1+(x/x_0_)^p)). The parameters are listed as follows:

CRP: there was good linearity in the range of 3 ng/ml–350 μg/ml, as shown in Figure 7A. The standard curve L (black square) covers a dynamic range of 3 ng/ml–0.781 μg/ml (LOD = 1 ng/ml), A_1_ = 0.11964, A_2_ = 3.05252, x_0_ = 0.06783, *p* = 0.73045, and *R* ^2^ = 0.9988. The standard curve H (red square) covers a dynamic range of 0.781–350 μg/ml, A_1_ = 3.0816, A_2_ = -0.04743, x_0_ = 17.27685, *p* = 0.58217, and *R* ^2^ = 0.9998. The value of indicators in the indicator curve (blue triangle) was calculated by combination with the standard curves L and H, which was set at the point of 1.745%.

PCT: there was good linearity in the range of 0.02–100 ng/ml, as shown in Figure 7B. The standard curve L (black square) covers a dynamic range of 0.02–50 ng/ml (LOD = 0.011 ng/ml), A_1_ = 0.02, A_2_ = 6.4, x_0_ = 1.80248, *p* = 0.97454, and *R* ^2^ = 0.9999. The standard curve H (red circle) covers a dynamic range of 2–100 ng/ml, A_1_ = 0.02571, A_2_ = -2.49554, x_0_ = 19.28237, *p* = 1.13747, and *R* ^2^ = 0.9999.

NGAL: there was good linearity in the range of 0.5–1,500 ng/ml, as shown in Figure 7C. The standard curve L (black square) covers a dynamic range of 0.5–100 ng/ml (LOD = 0.085 ng/ml), A_1_ = 0.02, A_2_ = 4, x_0_ = 29.53935, *p* = 0.66192, and *R* ^2^ = 0.9984. The standard curve H (red circle) covers a dynamic range of 50–1,500 ng/ml, A_1_ = 0.02, A_2_ = -2.4, x_0_ = 623.48545, *p* = 1.07164, and *R* ^2^ = 0.99682. The green line that connects the red square in Figure 7C shows the multivalued dose response, which demonstrates the ‘HOOK’ effect occurred in that value.

### 3.4 Measurement and Validation of the Chip for Multi-Detection

**FIGURE 8:**
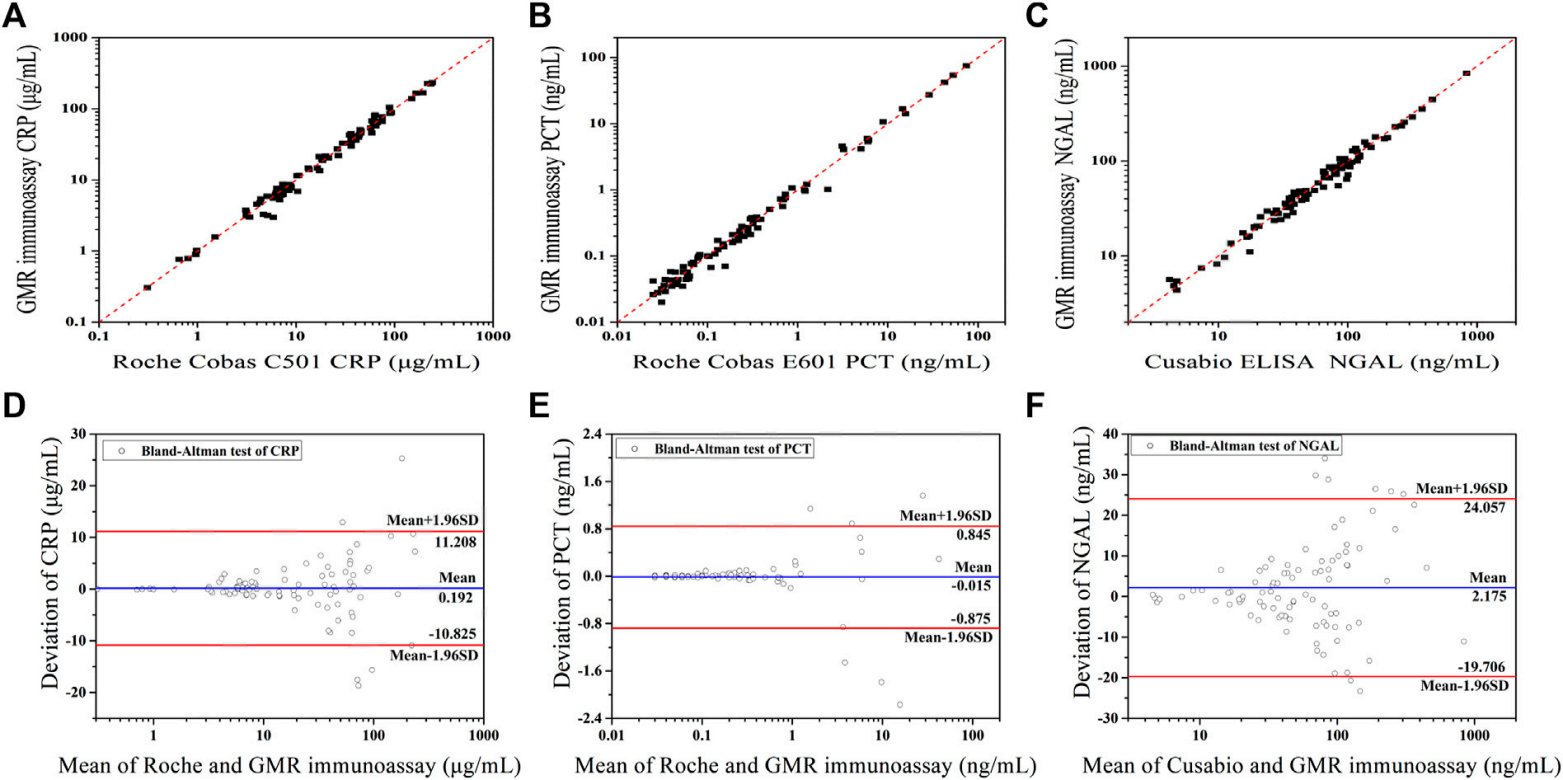
Method comparison between two assays: fitness analysis and Bland–Altman analysis of the agreement of CRP **(A,D)** (Adapted with permission from (Meng et al., 2021a) Copyright ^©^ 2021 Fanda Meng, etc. Published by American Chemical Society), PCT **(B,E),** and NGAL **(C,F)** detection between the GMR and commercialized assay.

The assay results obtained with the GMR detection system were validated against the commercialized assay (Figures 8A–C). Fitness analysis (*n* = 91) yielded the following equation for the data set, covering the entire concentration range.

The CRP assay results obtained with the GMR detection system were validated against the Roche Cobas C501 assay (Figure 8A). The PCT assay results obtained with the GMR detection system were validated against the Roche Cobas E601 assay (Figure 8B). The NGAL assay results obtained with the GMR detection system were validated against the Cusabio ELISA assay (Figure 8C).

For PCT detection, there were five samples with the concentration under the LOD (the results were reported <0.02 ng/ml), so only 86 samples were calculated.

All correlation coefficients were above 0.95(CRP: *r* = 0.9881, PCT: *r* = 0.9936, and NGAL: *r* = 0.9898). In Figures 8D–F, Bland−Altman plots of the relative differences between the data sets of the two compared assays were displayed. There were above 90% of points within the 95% confidence limit for all three biomarkers’ evaluation (CRP: 86 of 91 and 94.5%; PCT: 80 of 86 and 93.0%; and NGAL: 83 of 91 and 91.2%.).

There is no statistically significant bias between the GMR assay and commercialized assays, which shows the good potential clinical application of our established assay.

## 4 Conclusion

The multi-detection of combinational biomarkers in clinical settings with different abundance and dynamic ranges attracted wide attention in the area of the point-of-care test. Traditional assays are hard to balance the various sensitivities and different dynamic ranges of multi-biomarkers at the same time. Even though many combinations of biomarkers could better support the diagnosis of disease in clinical settings, they are hardly detected in one assay. Rapid quantification of multi-biomarkers in blood with a sensitive tool could, therefore, serve as a diagnostic of diseases, which also could be assessed for further clinical utility.

In the tandem GMR assay, two methods of expanding the dynamic range to cover the full clinically relevant concentration range were provided. The competitive assay was conjoined with a sandwich assay as the indicator, which could monitor the ‘HOOK’ effect. Therefore, the ‘HOOK’ effect curve was creatively ultimate as the standard curve, which provides an alternative method to analyze the bio target in the undiluted sample. In addition, the tandem operation of two pairs of antibodies with different affinities also effectively widens the dynamic range and guaranteed sensitivity.

Our portable GMR sensor in tandem realized the quantitative and multiplexed measurement of infection biomarker abundance. Three biomarkers (CRP, PCT, and NGAL) were simultaneously quantified. We combined two wide-range dynamic methods in a tandem GMR assay that realized the one-shot full-range quantification of multi-biomarkers of infection in clinical settings, which were not only with different abundance analytically but were also with totally different clinically relevant concentration ranges. In addition, simultaneous quantification of multi-biomarkers with both higher and lower abundance in one assay was achieved.

There is no interference in the combined detection of three markers and after an interfering substance is added, showing that the assay has good specificity. Validation with commercial assays revealed that the accuracy of the assay was excellent in clinical settings. The developed concept of tandem assay provides a convincing way to detect multi-analytes in one shot with the undiluted clinical sample, which also perfectly meet the requirement of clinical application, and the tandem assay would be explored to realize more simultaneous protein detection. Future studies would be involved to develop a unique set of biomarkers specific to other diseases and render the assay onto the current platform.

## Data Availability

The original contributions presented in the study are included in the article/Supplementary Material; further inquiries can be directed to the corresponding authors.
